# Management of Hypothyroidism in Pregnancy and Its Impact on Maternal and Perinatal Outcomes: A Single-Center Retrospective Cohort Study

**DOI:** 10.3390/life16030527

**Published:** 2026-03-22

**Authors:** Chinnu George Samuel, Asma Jamil, Mohamed Bashir, Hala Abdullahi, Ibrahim Ibrahim

**Affiliations:** 1 Clinical Trials Office, Sidra Medicine, Doha P.O. Box 26999, Qatar; cgeorgesamuel@sidra.org (C.G.S.); ajamil@sidra.org (A.J.); 2 Qatar Metabolic Institute, Endocrine Department, Hamad Medical Corporation, Doha Box 3050, Qatar; mbashir@hamad.qa; 3 Sidra Medicine, Weill Cornell Medical College-Qatar, Doha P.O. Box 24144, Qatar; habdullahi@sidra.org

**Keywords:** hypothyroidism, pregnancy, levothyroxine, maternal outcomes, TSH control, Qatar

## Abstract

Background: Hypothyroidism is one of the most common endocrine conditions during pregnancy and has been associated with poor obstetric and perinatal outcomes. There is still a lack of data from Middle Eastern populations, despite its clinical significance. This study aimed to evaluate thyroid management patterns during pregnancy and examine the association between thyroid function control and maternal and perinatal outcomes in women with hypothyroidism at a tertiary care center in Qatar. Methods: A retrospective cohort study including 379 pregnant women with hypothyroidism diagnosed between January 2019 and November 2022 was conducted at Sidra Medicine in Doha, Qatar. Based on trimester-specific Thyroid-stimulating hormone (TSH )reference values, participants were categorized as having adequately or inadequately controlled thyroid function. Data on obstetrics, biochemistry, and demographics were taken from electronic medical records (EMR). Statistical analyses were performed using chi-square tests for categorical variables and *t*-tests for continuous variables, with a significance threshold of *p* < 0.05. Results: Participants had a mean Body Mass Index (BMI) of 30.33 ± 6.14 kg/m^2^ and an average age of 32.65 ± 4.99 years; 54% of them were Qataris. Of the patients, 58.5% had positive thyroid antibodies and 55.7% had pre-gestational hypothyroidism. Women with pre-gestational hypothyroidism required significantly higher levothyroxine doses compared with those with gestational hypothyroidism (93.2 ± 47.5 mcg/day vs. 67.6 ± 30.1 mcg/day; *p* < 0.001). Treatment adjustment was demonstrated by the improvement in TSH normalization from 51.3% in the first trimester to 64.2% in the third trimester (*p* = 0.041). No significant associations were observed with pre-eclampsia, preterm delivery, hypertension, or placental abruption. However, women with normal third-trimester TSH had a higher prevalence of gestational diabetes mellitus (GDM) compared with those with elevated TSH (51.6% vs. 36.8%; *p* = 0.013). Conclusions: Appropriate trimester-specific monitoring and timely levothyroxine titration was associated with improved biochemical control without adverse maternal outcomes. Greater levothyroxine requirements in women with pre-gestational hypothyroidism emphasize the importance of early intervention. These findings highlight the potential benefit of structured thyroid monitoring and multidisciplinary care approaches in pregnancy and may help inform future regional clinical practice guidelines.

## 1. Introduction

Hypothyroidism is a common endocrine disorder in pregnancy, particularly in the Middle East, and is linked to adverse maternal and perinatal outcomes [1,2]. Subclinical hypothyroidism (SCH), defined as elevated TSH with normal free thyroxine, is frequently identified during pregnancy [1]. This study aims to clarify current management practices for hypothyroidism during pregnancy in the Middle East and to evaluate associated maternal and perinatal outcomes in this region. Untreated hypothyroidism increases the risk of perinatal complications such as spontaneous abortion, premature birth, placental abruption, stillbirth, low birth weight, poor neurodevelopment, and maternal complications like preeclampsia and gestational hypertension [3]. Appropriate thyroid hormone replacement during pregnancy minimizes these risks [2]. According to American Thyroid Association (ATA) guidelines, overt hypothyroidism is diagnosed when TSH exceeds trimester-specific reference intervals with decreased Free thyroxine(FT4) or when TSH is above 10.0 mIU/L, regardless of FT4. Levothyroxine remains the standard treatment, with early dose adjustment and regular TSH monitoring recommended [4].

Trimester-specific TSH goals are required because thyroid function changes during pregnancy. In our setting, the suggested upper TSH limits are: first trimester ≤ 2.5 mIU/L, second ≤ 3.0, and third ≤ 3.5, in line with international criteria. Maintaining TSH within these ranges using levothyroxine reduces maternal and neonatal complications [5]. Data from Qatar and the Gulf remain limited, but regional and international studies show high rates of thyroid dysfunction among pregnant women in the Middle East. A Qatar secondary care study found 3.47% had hypothyroidism, with one-third showing thyroid autoimmunity [6]. The “Qatar Birth Cohort Study” reported thyroid dysfunction in 19% and diabetes in 33% of pregnant women in Qatar [7]. Similarly, a meta-analysis of pregnant Arab women showed pooled prevalence estimates of subclinical hypothyroidism at ~20% (95% Confidence Interval(CI): 14–28%) and overt hypothyroidism at ~3% (95% CI: 1–8%) [8]. Variations in iodine intake, TSH reference ranges, healthcare access, and treatment adherence limit the direct application of international guidelines regionally. These factors, alongside differences in screening, diagnosis, and study design, likely explain the high SCH prevalence in Arab populations.

For this study, maintaining trimester-specific maternal TSH is considered adequate treatment. Given limited regional data and increasing recognition of thyroid dysfunction as a modifiable risk, we assess therapeutic approaches and maternal–perinatal outcomes of hypothyroidism in a Qatari cohort.

## 2. Study Aim and Hypothesis

This study’s primary goal is to assess differences in maternal and perinatal outcomes between individuals who achieve trimester-specific TSH targets in accordance with 2017 American Thyroid Association guidelines and those who do not. Maternal outcomes include preeclampsia, placental abruption, gestational diabetes and gestational hypertension, while perinatal outcomes include preterm birth, spontaneous abortion, and stillbirth. The study will investigate the hypothesis that pregnant women with hypothyroidism who achieve trimester-specific TSH targets have a lower incidence of unfavorable maternal and perinatal outcomes.

## 3. Materials and Methods

This single-center retrospective cohort study was conducted at Sidra Medicine in Doha, Qatar. The inclusion criteria comprised pregnant women who attended antenatal care at Sidra Medicine between January 2019 and November 2022, with a diagnosis of hypothyroidism and receiving thyroxine replacement therapy.

Eligible participants included women with either overt or subclinical hypothyroidism. Overt hypothyroidism was defined as a thyroid-stimulating hormone (TSH) level above the trimester-specific reference range with low free thyroxine (FT4) or when TSH is above 10.0 mIU/L, regardless of FT4, while subclinical hypothyroidism was defined as elevated TSH with normal FT4 levels. Women were included regardless of whether hypothyroidism was diagnosed prior to pregnancy (pre-gestational) or during pregnancy (gestational).

Exclusion criteria included patients with pre-gestational diabetes, chronic hypertension, multiple gestations, and congenital fetal anomalies. Additionally, records lacking essential information, such as delivery data, were excluded.

Electronic medical records of eligible women were reviewed. Based on trimester-specific TSH values, patients were classified as having adequately or inadequately controlled hypothyroidism. Adequate control was defined as TSH ≤ 2.5 mIU/L in the first trimester, ≤3.0 mIU/L in the second trimester, and ≤3.5 mIU/L in the third trimester. Values above these thresholds indicated inadequate control. These cutoffs follow the 2017 American Thyroid Association pregnancy guidelines and Sidra Medicine reference values.

GDM was diagnosed according to the International Association of Diabetes and Pregnancy Study Groups criteria, with universal 75 g oral glucose tolerance testing performed between 24 and 28 weeks’ gestation.

TSH was measured once per trimester during routine prenatal visits. At each assessment, values were compared with trimester-specific reference ranges to determine adequacy of control. Adequate control was defined as maintaining TSH within the reference range at each trimester assessment.

TSH, FT4, and Free triiodothyronine (FT3) were measured at Sidra Medicine’s central laboratory using automated immunoassays per manufacturer instructions (reference ranges: TSH 0.38–5.33 mIU/L, FT4 8.4–19.1 pmol/L, FT3 3.8–6.0 pmol/L). Anti-thyroid peroxidase antibodies (anti-TPO) were also measured via automated immunoassay (reference 0–10 IU/mL).

Data collected included maternal demographics (age, nationality, gravidity, parity, BMI), thyroid function tests (TSH, FT4, FT3, anti-TPO) at three prenatal visits, and obstetric history. Additional variables included maternal outcomes (gestational hypertension, diabetes), levothyroxine dose, hypothyroidism type (pre-gestational or gestational), and maternal and perinatal outcomes (preeclampsia, placental abruption, spontaneous abortion, stillbirth, preterm labor, delivery method, gestational age, and neonatal birth weight).

Associations between thyroid control and maternal and perinatal outcomes were assessed using chi-square tests for categorical variables and independent *t*-tests for continuous variables. Adjusted odds ratios with 95% confidence intervals were reported when feasible; estimates for rare outcomes were not reported due to instability. Comparisons of thyroid status across trimesters were descriptive and exploratory, and formal repeated-measures modeling was not performed due to limited longitudinal data. Baseline characteristics were summarized descriptively. Additionally, an exploratory multivariable logistic regression was conducted to identify potential risk factors for GDM; these analyses are considered hypothesis-generating given the sample size and limited precision in effect estimates. TSH was analyzed as a categorical variable based on trimester-specific reference ranges (“normal” vs. “high”).

Because third-trimester TSH showed the strongest univariate association with GDM, it was included in the multivariable logistic regression model. Model calibration was assessed using the Hosmer-Lemeshow goodness-of-fit test, with a non-significant *p*-value (>0.05) indicating adequate fit. Collinearity was evaluated via variance inflation factors (VIF), with VIF > 5 considered indicative of problematic collinearity.

No prior power calculation was performed due to the retrospective design and available sample size. With 379 participants, the study had 80% power at a 5% significance level to detect moderate to large differences (≈15–20%) in binary maternal outcomes (e.g., GDM, preeclampsia). Results were reported descriptively using proportions and *p*-values.

**Figure 1:**
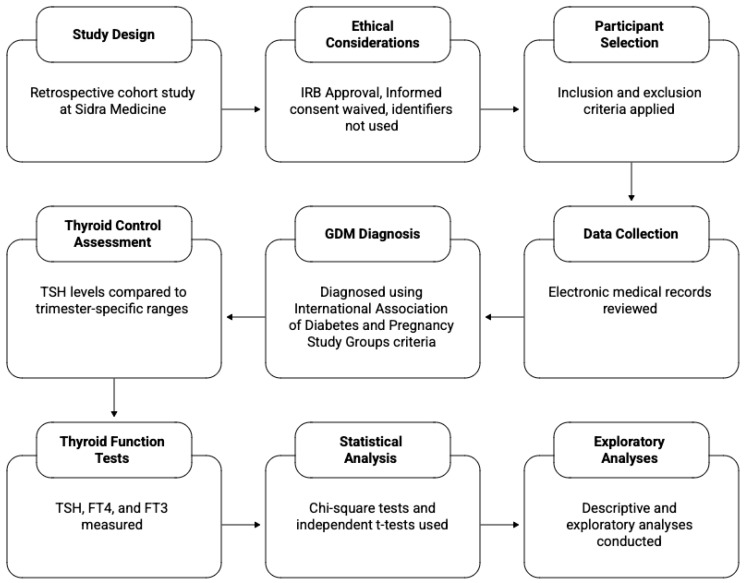
Hypothyroidism in Pregnancy Study Methodology.

No patient identifiers were used, and informed consent was waived because the study used only pre-existing records without direct patient contact. Figure 1 outlines the study design, participant selection, data collection, analysis, and ethics. A normal-thyroid comparison group was not available; analyses compared only adequately versus inadequately treated hypothyroidism.

## 4. Results

The study included 379 pregnant women with hypothyroidism. Baseline characteristics are summarized in Table 1. Participants had a mean age of 32.7 ± 5.0 years (range 19–46) and were mostly overweight or obese, with a mean BMI of 30.3 ± 6.1 kg/m^2^ and weight of 78.6 ± 15.1 kg. Qatari nationals comprised 54.1% of the cohort, followed by Asians (16.9%), other nationalities (17.2%), and Arabs (11.9%). Pre-gestational hypothyroidism was present in 55.7%, and gestational hypothyroidism in 44.3%. Anti-TPO antibodies were elevated in 58.5% of participants.

Table 2 compares thyroid function and treatment between gestational and pre-gestational hypothyroidism. Women with gestational hypothyroidism had higher baseline TSH levels (3.22 ± 2.30 mIU/L) than pre-gestational cases (2.65 ± 1.83 mIU/L; *p* = 0.015), reflecting ongoing levothyroxine therapy in the latter group. Pre-gestational patients required higher levothyroxine doses (93.2 ± 47.5 vs. 67.6 ± 30.1 mcg/day; *p* < 0.001). FT4 levels were slightly higher in the gestational group at baseline (11.87 ± 4.20 vs. 11.09 ± 3.65 pmol/L; *p* = 0.048). No significant differences were observed at the last thyroid assessment or in gestational age at delivery.

Pregnancy outcomes according to first- and third-trimester TSH status are shown in Table 3. No significant differences were observed in the first trimester for GDM, hypertension, preeclampsia, placental abruption, spontaneous abortion, or preterm birth. In the third trimester, women with normal TSH had a higher prevalence of GDM than those with elevated TSH (51.6% vs. 36.8%; *p* = 0.013), while no differences were noted for other outcomes. Odds ratios were calculated only for GDM in the multivariable model due to low event numbers for other outcomes.

Multivariable logistic regression, including maternal age, gravidity, parity, BMI, nationality, and third-trimester TSH, identified factors independently associated with GDM (Table 4). Normal third-trimester TSH was independently associated with higher odds of GDM (OR 1.70, 95% CI 1.04–2.78; *p* = 0.035). Model fit was adequate (Hosmer-Lemeshow χ^2^ = 296.14, *p* = 0.374), and no collinearity was detected (all VIF < 5).

## 5. Discussion

In this cohort of pregnancies complicated by hypothyroidism, most maternal outcomes were not significantly influenced by thyroid control status. TSH targets for each trimester were <2.5 mIU/L in the first, <3.0 mIU/L in the second, and <3.5 mIU/L in the third trimester. By the third trimester, 64.2% of women achieved these targets, reflecting effective levothyroxine titration and monitoring. This aligns with prior studies showing that optimized biochemical thyroid management does not adversely affect obstetric outcomes [9]. Dynamic, trimester-specific dose escalation is supported by the observation that women with pre-gestational hypothyroidism required higher levothyroxine doses than those with gestational hypothyroidism [10,11,12].

Women with normal third-trimester TSH had a higher prevalence of GDM compared with those with elevated TSH. This association is unlikely to reflect a direct causal effect of thyroid normalization and may instead result from residual confounding or increased clinical surveillance [13,14,15]. Although subtle alterations in thyroid hormones, such as low FT4 or altered FT3/FT4 ratios, have been proposed to affect glucose metabolism, the mechanisms remain speculative and warrant cautious interpretation [13,14].

Thyroid status did not significantly impact outcomes such as spontaneous abortion, preterm birth, placental abruption, hypertensive disorders, or preeclampsia, likely due to their low incidence [16,17]. Mildly elevated first-trimester TSH (2.5–4.0 mIU/L) has also been shown not to adversely affect pregnancy outcomes in TPOAb-negative women, suggesting that early pregnancy upper TSH limits may safely be raised in this population [18]. While levothyroxine treatment is generally beneficial in biochemical hypothyroidism and may improve live birth rates in antibody-positive euthyroid women, subgroup analyses were limited due to incomplete TPOAb data [19,20,21,22,23].

These findings emphasize the importance of ongoing trimester-specific dose optimization rather than relying on a single early TSH assessment [24,25]. Pre-gestational hypothyroidism and thyroid antibody positivity are common in Gulf Cooperation Council countries, despite sufficient iodine intake [26]. Accordingly, adaptive levothyroxine titration is preferable to fixed-dose supplementation, particularly in populations with high baseline thyroid disease and GDM risk [27]. Further research with matched euthyroid controls is needed to confirm these results, as the absence of a normal-thyroid comparison group limits assessment of relative maternal and perinatal risks.

In summary, timely, trimester-specific levothyroxine adjustment is crucial in managing hypothyroidism during pregnancy. Women with pre-gestational hypothyroidism required higher doses, highlighting the need for early detection and individualized therapy. The observed association between third-trimester thyroid status and GDM underscores the complex interplay between thyroid function and glucose metabolism but should be interpreted as exploratory; reverse causality cannot be excluded, as GDM diagnosis and monitoring may influence thyroid testing.

## 6. Strengths

This study provides valuable regional data from Qatar on the management of hypothyroidism in pregnancy, addressing an important gap in Middle Eastern literature. The relatively large cohort and use of electronic medical records ensured standardized biochemical data collection and minimized recall bias. The single-center design promoted consistency in laboratory reference ranges, thyroid monitoring, and levothyroxine titration practices. Evaluation across all three trimesters allowed assessment of dynamic dose adjustments and demonstrated progressive improvement in TSH control. The use of guideline-based trimester-specific TSH targets and exploratory multivariable analysis further strengthened the clinical relevance of the findings.

## 7. Limitations

This retrospective single-center study has limited generalizability and cannot establish causality. The single-center design ensured consistent thyroid monitoring, reference ranges, and clinical care. Incomplete TPO antibody testing restricted subgroup analyses, and the lack of iodine, vitamin D, and neonatal data limits interpretation of autoimmune and metabolic effects. The absence of a euthyroid control group limits direct comparisons, though internal analyses allowed evaluation of treatment effectiveness. Inclusion of both overt and subclinical hypothyroidism may increase clinical heterogeneity. Extraction from multiple EMR modules prevented reliable determination of screened records, introducing potential selection bias.

Trimester-specific TSH thresholds, rather than continuous measures, may reduce sensitivity to subtle associations; future studies could consider continuous or quantile-based modeling. Most FT4 values were within reference limits, limiting distinction between compensated and overtreated thyroid conditions. Not all participants underwent early GDM screening, and the higher observed GDM frequency with normalized third-trimester TSH should be interpreted cautiously due to potential residual confounding, treatment effects, reverse causality, or early metabolic programming.

Limited statistical power from the descriptive, retrospective design and low event rates constrains adjusted analyses. Observed associations are therefore descriptive rather than causal.

## 8. Future Directions

Future studies should prospectively track thyroid dynamics from preconception through the postpartum period. Including comprehensive neonatal outcomes would allow assessment of long-term developmental effects. Molecular and genetic studies could clarify regional risk factors for thyroid autoimmunity in the Gulf. Comparative trials of fixed-dose versus adaptive LT4 regimens may optimize treatment strategies. Finally, establishing a regional thyroid-in-pregnancy registry could enhance research collaboration, improve care quality, and inform public health policy.

## 9. Conclusions

Timely LT4 titration and trimester-specific monitoring supports adaptive thyroid management during pregnancy, with maximum normalization achieved by the third trimester. Women with pre-gestational hypothyroidism require higher LT4 doses, highlighting the importance of early detection and individualized dosing. The observed association between third-trimester thyroid status and GDM reflects the complex interplay between thyroid function and glucose metabolism and should be considered descriptive and hypothesis-generating.

## Figures and Tables

**Table 1:** Demographic, Clinical, and Diagnostic Characteristics (N = 379).

Variable	Mean ± SD	Range
Age (years)	32.65 ± 4.99	19–46
Weight (kg)	78.57 ± 15.05	38–130
BMI (kg/m^2^)	30.33 ± 6.14	14.66–49.64
Gravidity	3.33 ± 1.98	1–9
Parity	1.74 ± 1.60	0–9

**Table 2:** Thyroid Function and Treatment by Hypothyroidism Type.

Variable	Normal Range	Gestational Hypothyroidism (Mean ± SD)	Pre-Gestational Hypothyroidism (Mean ± SD)	*p*-Value
TSH (first)	≤2.5 mIU/L	3.22 ± 2.30	2.65 ± 1.83	0.015
FT4 (first)	8.4–19.1 pmol/L	11.87 ± 4.20	11.09 ± 3.65	0.048
TSH (last)	≤3.5 mIU/L	2.71 ± 2.51	2.32 ± 2.25	0.11
FT4 (last)	8.4–19.1 pmol/L	11.54 ± 3.28	11.12 ± 3.19	0.231
LT4 Dose (mcg/day)	-	93.2 ± 47.5	67.6 ± 30.1	<0.001
Gestational Age (weeks)	-	38.57 ± 2.31	37.85 ± 4.36	0.081

**Table 3:** Maternal and Perinatal Outcomes by TSH Status.

Outcome	First Trimester Normal TSH (%)	First Trimester High TSH (%)	*p*-Value	Third Trimester Normal TSH (%)	Third Trimester High TSH (%)	*p*-Value
GDM	45.7	48.4	0.23	51.6	36.8	0.013
Hypertension	4.5	5.8	0.65	5.5	4.3	0.71
Preeclampsia	1.0	1.2	0.78	2.0	0.6	0.35
Placental Abruption	2.0	2.3	0.87	3.4	1.2	0.33
Spontaneous Abortion	0.8	0.7	0.90	1.6	1.7	0.89
Preterm Birth	9.7	8.9	0.20	11.9	11.5	0.19

**Table 4:** Multivariable Logistic Regression Analysis for GDM.

Variable	OR	Std. Err.	*p*	95% CI
Third Trimester TSH	1.70	0.43	0.035	1.03–2.78
Age	1.01	0.03	0.730	0.95–1.07
Gravidity	1.16	0.13	0.200	0.92–1.46
Parity	1.00	0.14	1.000	0.74–1.30
BMI	0.99	0.02	0.980	0.96–1.03
Nationality	1.05	0.12	0.660	0.84–1.31

## Data Availability

The data presented in this study are not publicly available due to patient privacy and ethical restrictions. The clinical datasets contain sensitive patient information and are protected under the regulations of Sidra Medicine and the Institutional Review Board. De-identified data may be made available from the corresponding author upon reasonable request and subject to approval by the Sidra Medicine Institutional Review Board.

## Abbreviations

The following abbreviations are used in this manuscript:

ATA	American Thyroid Association
BMI	Body Mass Index
CI	Confidence Interval
VIF	(Variance inflation factors
EMR	Electronic Medical Record
FT3	Free Triiodothyronine
FT4	Free Thyroxine
GCC	Gulf Cooperation Council
GDM	Gestational Diabetes Mellitus
HTN	Hypertension
LPI	Lead Principal Investigator
LT4	Levothyroxine
MoPH	Ministry of Public Health
NICU	Neonatal Intensive Care Unit
OH	Overt Hypothyroidism
PET	Preeclampsia/Pregnancy-Induced Hypertension
PIH	Pregnancy-Induced Hypertension
RCT	Randomized Controlled Trial
SCH	Subclinical Hypothyroidism
SD	Standard Deviation
TSH	Thyroid-Stimulating Hormone
TPOAb	Thyroid Peroxidase Antibody
